# 

**Published:** 1918-09-21

**Authors:** 


RATES OF SUBSCRIPTION:

Twelve months, 10/10; Six months, 5/5; Three months, 2/9, post free to any address in the United Kingdom. Abroad, Twelve months, 13/-; Six months, 6/6; Three months, 3/3, post free. **THE HOSPITAL "Index" to Volume LXI., October 1917 to March 1918, is now ready, price 3½d. per copy, post free.** Address The Manager, The Hospital, "The Hospital" Building, 28/29 Southampton Street, Strand, London, W.C. 2.

### Theatres and "Our Day."

At the request of the British Red Cross Society and the Order of St. John, the Entertainments Industry Committee, under the chairmanship of Mr. Oswald Stoll, and including well-known representatives of theatres, music-halls, cinematographs, actors, variety artists, etc., is organising a great united effort on behalf of "Our Day, 1918." It is intended and hoped to give evidence of the power that the entertainment industry wields in charitable work. Collections will be made weekly from the staffs of theatres, etc., from September 30 to October 24, and from audiences during the same period. The address of the committee is Hôtel Windsor, Victoria Street, S.W., and all connected in any way with places of amusement, who would like to help, are asked to communicate with the honorary secretary.

### Hospital and Institutional Results.

**St. Thomas's Hospital.**—The annual expenditure of the hospital has now reached the substantial total of £130,000. The average cost of each in-patient per week in 1917 was £2 7s. 6d., as compared with £2 4s. 6d. in the previous year. £33,907 was received from the War Office in respect of the military section. The annual subscription list—£1,394—is very small for such a large institution.

**St. Mary's Hospital.**—The annual report is abbreviated for reasons of economy. The number of out-patients treated has fallen steadily from 53,245 in 1912 to 30,306 in 1917. The report includes a most interesting table of prices paid for provisions and medical and surgical stores during the war.

**Royal Hospital for Sick Children, Glasgow.**—There are 220 pages of closely-printed matter in the annual report. In view of increased public interest in infant welfare and mortality the directors wisely bring out what is being done for the children by the voluntary hospitals. It is to be noted, however, that the military authorities continue to requisition four wards for the use of sick and wounded officers. It is to be hoped that this diversion of the accommodation of the hospital from its proper use will as early as possible cease, and that the hospital for children and women will be the first to be relieved of what was intended to be only a temporary use while hospital beds were being prepared elsewhere.

**British Hospital for Mothers and Babies, Woolwich.**—The institution is making the best of the delay in getting on with the permanent hospital by strengthening the building fund and by utilising the three-acre site for the raising of vegetables.

**London Orphan Working School, Watford.**—The excess of expenditure over income for the two years ending December 1917 was £5,857. Orphanages do not appear to attract public sympathy during the war as much as they deserve, and it is hoped that the joint appeal issued by the Lord Mayor will relieve the financial situation.

